# Expression and assembly of largest foreign protein in chloroplasts: oral delivery of human FVIII made in lettuce chloroplasts robustly suppresses inhibitor formation in haemophilia A mice

**DOI:** 10.1111/pbi.12859

**Published:** 2017-12-07

**Authors:** Kwang‐Chul Kwon, Alexandra Sherman, Wan‐Jung Chang, Aditya Kamesh, Moanaro Biswas, Roland W. Herzog, Henry Daniell

**Affiliations:** ^1^ Department of Biochemistry School of Dental Medicine University of Pennsylvania Philadelphia PA USA; ^2^ Department of Pediatrics University of Florida Gainesville FL USA

**Keywords:** bioencapsulation, human blood clotting factor, oral delivery, antibody suppression

## Abstract

Inhibitor formation is a serious complication of factor VIII (FVIII) replacement therapy for the X‐linked bleeding disorder haemophilia A and occurs in 20%–30% of patients. No prophylactic tolerance protocol currently exists. Although we reported oral tolerance induction using FVIII domains expressed in tobacco chloroplasts, significant challenges in clinical advancement include expression of the full‐length CTB‐FVIII sequence to cover the entire patient population, regardless of individual CD4^+^ T‐cell epitope responses. Codon optimization of FVIII heavy chain (HC) and light chain (LC) increased expression 15‐ to 42‐fold higher than the native human genes. Homoplasmic lettuce lines expressed CTB fusion proteins of FVIII‐HC (99.3 kDa), LC (91.8 kDa), C2 (31 kDa) or single chain (SC, 178.2 kDa) up to 3622, 263, 3321 and 852 μg/g in lyophilized plant cells, when grown in a cGMP hydroponic facility (Fraunhofer). CTB‐FVIII‐SC is the largest foreign protein expressed in chloroplasts; despite a large pentamer size (891 kDa), assembly, folding and disulphide bonds were maintained upon lyophilization and long‐term storage as revealed by GM1‐ganglioside receptor binding assays. Repeated oral gavages (twice/week for 2 months) of CTB‐FVIII‐HC/CTB‐FVIII‐LC reduced inhibitor titres ~10‐fold (average 44 BU/mL to 4.7 BU/mL) in haemophilia A mice. Most importantly, increase in the frequency of circulating LAP‐expressing CD4^+^ CD25^+^FoxP3^+^ Treg in tolerized mice could be used as an important cellular biomarker in human clinical trials for plant‐based oral tolerance induction. In conclusion, this study reports the first clinical candidate for oral tolerance induction that is urgently needed to protect haemophilia A patients receiving FVIII injections.

## Introduction

The serine protease factor IX (FIX) and its cofactor, factor VIII (FVIII), circulate in the blood in their inactive form. Upon their activation, they form an enzymatic complex that is a critical component of the coagulation cascade, which promotes blood clot formation, thereby aiding in vascular injury repair. However, approximately one in 5000 boys worldwide is born with the bleeding disorder haemophilia resulting from mutations on the X chromosome. The majority of patients lack FVIII activity (haemophilia A), while the remainder lack FIX (haemophilia B). Currently, treatment of haemophilia is primarily based on replacement therapy by intravenous infusion of recombinant or plasma‐derived factor concentrate. Unfortunately, 20%–30% of haemophilia A patients develop inhibitory antibodies (‘inhibitors’) against FVIII that severely complicate treatment and increase morbidity and mortality risk (Lai and Lillicrap, 2017). These inhibitors typically develop in young children during the first 50 days of exposure to the therapeutic FVIII protein. Immune tolerance induction (ITI) protocols are able to eradicate inhibitors with a success rate of approximately 60% (Kempton and Meeks, 2014). ITI utilizes daily high‐dose intravenous FVIII infusions and can take months or even years to complete, often posing costs of >$1 000 000. During that time, bypass agents can restore haemostasis, but require careful dosing to prevent thrombosis, and increased potential for morbidity and mortality remain during that time (Rocino *et al*., 2017). Progress has been made in defining the risk factors for inhibitor formation. However, there are no prophylactic immune tolerance protocols to prevent inhibitor formation. Similar challenges exist in replacement therapies for other genetic diseases as a result of antidrug antibody formation, such as for the lysosomal storage disorder Pompe disease, prompting the prolonged use of immune‐suppressive drugs (Doerfler *et al*., 2016; Elder *et al*., 2013).

Although there are a number of recent innovative approaches to induce FVIII‐specific immune tolerance, developing a clinically feasible protocol that is acceptable for use in paediatric patients and avoids nonspecific immune suppression remains a challenge (Batsuli *et al*., 2016; Wang *et al*., 2016). Oral antigen administration represents a potential solution to this problem (Kuhn and Weiner, 2016; Rezende and Weiner, 2017; Wang *et al*., 2013). The gut immune system has evolved mechanisms to prevent pathogenic immune response to food antigens. Therefore, orally administered antigens, similar to food antigens, may be taken up by the small intestine's immune system, resulting in an immune regulatory response. This concept has recently been successfully employed in children with severe food allergies (Hamad and Burks, 2017). A perhaps greater challenge is to adapt this concept to therapeutic proteins, which will have to be manufactured in a cost‐efficient manner, formulated to be protected from degradation before reaching the gut, and effectively targeted to the small intestine's immune system in order to achieve an immune regulatory response that is capable of systemically suppressing immune responses to the specific antigen. We developed a platform to accomplish these goals by expression of protein antigens (fused to transmucosal carrier proteins) in chloroplasts of crop plants. These plants achieve expression of effective antigen doses, while also providing natural bioencapsulation by the cell wall and avoiding the need for expensive protein purification (Daniell *et al*., 2016a,b; Kwon and Daniell, 2016; Kwon *et al*., 2013a). Importantly, this concept has been successfully applied in murine models of haemophilia A and B and Pompe disease, and even in a large (canine) model of haemophilia B, illustrating translatability to other species (Herzog *et al*., 2017; Sherman *et al*., 2014; Su *et al*., 2015a,b; Verma *et al*., 2010).

Using a murine model of haemophilia A, we previously demonstrated in a proof‐of‐principle study that oral delivery of FVIII domains bioencapsulated in frozen leaf cells of transplastomic tobacco suppresses inhibitor formation. This was accomplished using the heavy chain and C2 domain of B domain‐deleted (BDD) FVIII (one of the therapeutic FVIII variants used in replacement therapy for haemophilia A) as tolerogenic antigens. However, expression of the full‐length protein antigen is needed to cover the entire patient population, regardless of individual CD4^+^ T‐cell epitopes that induce inhibitory antibodies against FVIII. This requires optimization of chloroplast expression of large human blood proteins in edible leaves, evaluation of their stability at room temperature, protection of antigens in the stomach from acids and enzymes, successful release of antigens in the gut lumen and delivery to the immune system (Daniell *et al*., 2015, 2016b; Kwon and Daniell, 2015, 2016).

Chloroplasts express small human blood proteins very efficiently. CTB‐fused human proinsulin was expressed in chloroplasts 53%–72% of total leaf protein (22.5 kDa – Boyhan and Daniell, 2011; Ruhlman *et al*., 2010). Other smaller human therapeutic proteins including human insulin‐like growth factor‐1 (25 kDa – Daniell *et al*., 2009), human interferon alpha 2B (21.5 kDa – Arlen *et al*., 2007), TGF‐Beta (13 kDa – Gisby *et al*., 2011), were also expressed at high levels in chloroplasts. In contrast, larger native human gene like CTB‐fused human coagulation factor IX (59.2 kDa) was expressed only up to 0.56% of total lettuce leaf protein (Su *et al*., 2015b). Likewise, native coagulation factor VIII (FVIII) heavy chain (HC, 86.4 kDa) was expressed <0.05% of the total leaf proteins (Kwon *et al*., 2016). Such low levels of expression of large proteins are not due to low abundance of transcripts because it is heavily compensated by the high copy number of chloroplast genomes. We recently developed a codon optimizer program to increase transgene expression in lettuce and tobacco chloroplasts utilizing a wealth of newly sequenced chloroplast genomes. While the removal of rare codons is very important, replacing those with only the most highly used *psbA* codons decreased translation efficiency because of transfer RNA (tRNA) and other limitations (Kwon *et al*., 2016). Translation enhancement of human genes was achieved by replacement of rare codons following the hierarchy of the highly expressed chloroplast *psbA* gene. Therefore, there is a greater need to optimize expression of large human genes in chloroplasts for advancing them to the clinic.

In addition to challenges in expression of large human genes in chloroplasts, there are major obstacles for oral delivery of protein drugs. When orally delivered, protein drugs are degraded by acid and pepsin in the stomach, their bioavailability is dramatically reduced. In addition, proteins encounter several permeability barriers in the gut epithelium. Therefore, current protein drug delivery system is totally dependent on injections, which has been a major hurdle for lowering production cost, enhancing protein stability and increasing patient compliance. To address these issues, several other delivery methods including nasal, buccal, pulmonary, ocular, rectal and transdermal routes have been investigated with limited success (Kwon *et al*., 2013a). Attempts to enhance efficiency of oral delivery system have been made using protease inhibitors, mucoadhesion and absorption enhancers, and delivery vehicles formulated with emulsions, liposomes, microspheres and nanoparticles (Kwon *et al*., 2013a). Although many oral formulations have been developed to increase permeability and/or bioavailability of protein drugs, further studies are required for their dose‐dependent delivery (Kwon and Daniell, 2016).

Aforementioned challenges can be addressed by bioencapsulation of protein drugs within plant cells because plant cell wall is not digested by enzymes and low pH in the stomach. Upon reaching the intestine, plant cell wall is digested by enzymes secreted from commensal bacteria including Bacteroidetes and Firmicutes (Flint *et al*., 2012; Koropatkin *et al*., 2012). The released proteins cross the epithelial physical barrier with the help of fusion tags to reach circulation or immune system (Xiao *et al*., 2016). Also, freeze‐dried plant cells can be stored at ambient temperature for several years, preserving folding and assembly of protein drugs with no loss of functionality (Daniell *et al*., 2016a,b; Herzog *et al*., 2017; Su *et al*., 2015b). In addition, the removal of water from plant cells during lyophilization enhances concentration of protein drugs based on weight and facilitates oral drug delivery. Furthermore, cultivation of plants in confined optimal growth conditions in current Good Manufacturing Processes (cGMP) hydroponic system facilitates clinical development of plant‐made biopharmaceuticals. Application of this system has been evaluated in animal models of various human diseases including Alzheimer's (Kohli *et al*., 2014), pulmonary hypertension (Shenoy *et al*., 2014), diabetes (Boyhan and Daniell, 2011; Kwon *et al*., 2013b; Ruhlman *et al*., 2007) and retinopathy (Shil *et al*., 2014). Most importantly, this approach is suitable for induction of oral tolerance to therapeutic proteins used in replacement therapy of genetic diseases, as demonstrated in Pompe disease (Su *et al*., 2015a) and haemophilia (Herzog *et al*., 2017; Sherman *et al*., 2014; Wang *et al*., 2015) by suppression of immunoglobulin formation.

In this study, we achieved expression of the CTB fusion proteins of FVIII light chain (LC), C2 domain (C2) and the entire BDD human FVIII protein (SC) in lettuce chloroplasts to levels required for clinical translation and produced them in a cGMP facility at Fraunhofer USA. CTB‐FVIII‐SC is the largest foreign protein expressed so far in chloroplasts and the size of the assembled pentameric structure is 891 kDa; this is stable in lyophilized cells when stored at ambient temperature for several months with proper folding, disulphide bonds and assembly. All four CTB‐FVIII proteins (HC, LC, SC, C2) expressed in lettuce chloroplasts were tested for the first time in animal studies. Furthermore, we show that low oral antigen doses in lyophilized lettuce cells are effective in suppression of inhibitor formation against FVIII injections and identified a cellular biomarker of oral tolerance induction in peripheral blood cells useful in human clinical trials. These accomplishments augur well for advancing the first clinical candidate for oral tolerance induction to meet this unmet and urgent need for protecting haemophilia A patients receiving FVIII injections.

## Results

### Codon optimization and evaluation of human FVIII expression in *E. coli*

In our previous study (Sherman *et al*., 2014), expression level of the native human FVIII‐HC gene in tobacco chloroplasts was very low and tobacco system is not suitable for clinical studies. To enhance expression level of FVIII proteins in lettuce chloroplasts and to cover the entire FVIII sequence for the improved induction of oral tolerance, the genes for the heavy chain (HC) and light chain (LC) were codon‐optimized on the basis of the analysis of the most highly expressed *psbA* genes (Kwon *et al*., 2016). Rare codons were replaced by highly preferred codons and the hierarchy of codon usage of the chloroplast *psbA* gene was matched during the codon optimization process.

In codon‐optimized genes (Figure S1), 406 of 754 codons for FVIII‐HC including B domain (14 codons) and 445 of 684 codons for FVIII‐LC were optimized, which increased AT content from 56% to 62%. To obtain full‐length FVIII and B domain‐deleted sequence (FVIII‐BDD single chain, SC), the separately codon‐optimized FVIII‐HC and FVIII‐LC were fused by assembly PCR. In case of the cholera nontoxic subunit B (CTB), used as a transmucosal carrier protein, the native sequence was employed due to its high‐level expression in plant chloroplasts (Boyhan and Daniell, 2011; Ruhlman *et al*., 2010). The native human FVIII‐C2 gene was expressed at very high levels in tobacco chloroplasts (Sherman *et al*., 2014), more than adequate for translation, and therefore, this sequence was moved from tobacco to lettuce chloroplast vectors.

**Figure 1:**
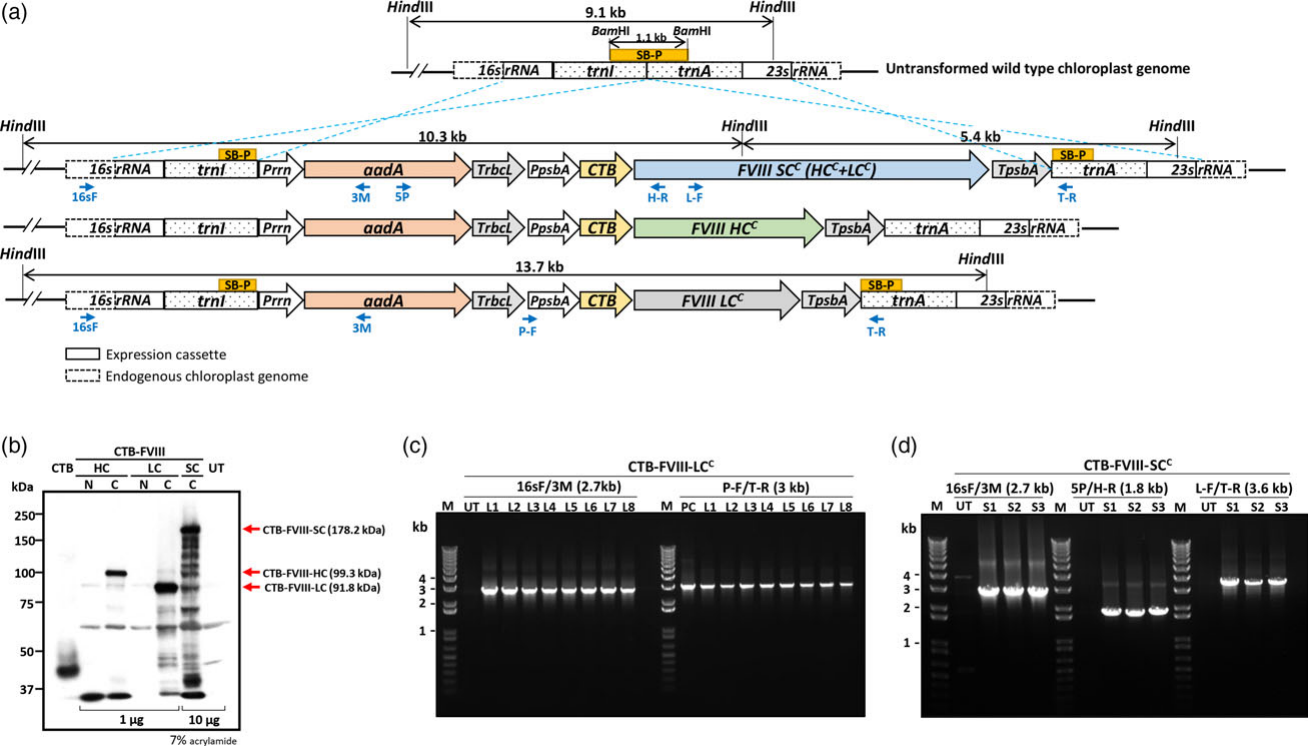
Chloroplast expression vectors, evaluation of expression in *E. coli* and CTB‐FVIII integration into chloroplast genomes. (a) Chloroplast expression cassette containing CTB‐fused codon‐optimized FVIII single chain (SC^C^), heavy chain (HC^C^) and light chain (LC^C^). P*rrn*, rRNA operon promoter; *aadA*, aminoglycoside 3′‐ adenylyltransferase gene; P*psbA*, promoter; and 5′‐UTR of *psbA* gene; *CTB*, native coding sequence of cholera nontoxic B subunit; *FVIII‐SC* ^*C*^, a fusion form of codon‐optimized FVIII heavy chain (*HC* ^*C*^ including 14 amino acids from B domain) and light chain (*LC* ^*C*^); T*psbA*, 3′‐UTR of the *psbA* gene; *trn*I, isoleucyl‐tRNA; *trn*A, alanyl‐tRNA; *16s* and *23s rRNA*, 16s and 23s ribosomal RNA, respectively. Southern blot – probe (SB‐P, 1.1 kb) generated from the flanking sequence; genomic DNA was digested by *Hind*III. (b) Western blot assays for CTB‐FVIII protein expression of native (N) or codon‐optimized (C) for HC, LC and SC in *E. coli*. Protein size: CTB‐FVIII‐SC, 178.2 kDa; CTB‐FVIII‐HC, 99.3 kDa; CTB‐FVIII‐LC, 91.8 kDa. (c) and (d) are PCR analysis of the integration of *CTB‐FVIII‐LC* ^*C*^ and of *CTB‐FVIII‐SC* ^*C*^ of transgenic lettuce. The primers used for PCR analysis are shown in A. UT, untransformed wild‐type DNA; L1–L8, eight independent CTB‐FVIII‐LC^C^ transplastomic lines; S1–S3, three independent CTB‐FVIII‐SC^C^ transplastomic lines; M, DNA 1kb plus marker.

In the chloroplast vector expression cassette (Figure 1a), the native CTB sequence is followed by hinge (Gly‐Pro‐Gly‐Pro) and furin cleavage site (Arg‐Arg‐Lys‐Arg‐Ser‐Val, Duckert *et al*., 2004) to avoid any steric hindrance and to facilitate efficient release of the fused FVIII proteins (Figure S1). The CTB‐fused FVIII genes are driven by strong *psbA* promoter/5ʹ‐UTR, which is most widely used for high expression of transgenes in chloroplasts (Daniell *et al*., 2016b), and the transcripts of the fusion genes are stabilized by the *psbA* 3ʹ‐UTR. To ensure site‐specific integration of expression cassettes into lettuce chloroplast genomes by double homologous recombination, 3′ half of *16s rRNA*,* trn*I genes (2098 bp) and *trn*A, 5′ half *23s rRNA* (2047 bp) flanked both ends of the cassettes. Aminoglycoside‐3′‐adenylyltransferase selectable marker gene (*aadA*) is driven by ribosomal RNA promoter (P*rrn*).

After construction of the codon‐optimized synthetic genes into chloroplast transformation vectors, translation efficiency of the synthetic genes over native sequences was tested in *E. coli* because of similarity of both expression systems in transcription and translation (Brixey *et al*., 1997). All three CTB fusion proteins, FVIII‐HC (99.3 kDa), FVIII‐LC (91.8 kDa) and FVIII‐SC (178.2 kDa), were detected at the expected sizes (Figure 1b). According to the immunoblot results (Figure 1b), at equal loading amount (1 μg of total protein), the corresponding polypeptides of CTB‐FVIII‐HC and CTB‐FVIII‐LC were barely detectable. In contrast, expression level of codon‐optimized CTB‐FVIII‐HC and CTB‐FVIII‐LC genes in *E. coli* increased 15‐ to 42‐fold. These results indicate that codon optimization system is very effective in improving FVIII expression.

### Creation and evaluation of homoplasmic transplastomic lettuce lines expressing codon‐optimized human FVIII genes

The CTB‐FVIII gene constructs were delivered into the lettuce chloroplasts by biolistic particle delivery system. Transplastomic plants were selected on spectinomycin and examined by PCR amplification of genomic DNA (Figure 1). The PCR results of all independent lettuce lines examined confirmed site‐specific integration of CTB‐FVIII‐LC (Figure 1c) and CTB‐FVIII‐SC (Figure 1d). The specific integration of the FVIII expression cassettes into lettuce chloroplast genome was evaluated using primers: 16sF, annealed to the endogenous *16s rRNA* gene outside the expression cassettes, and 3M, annealed to the *aadA* gene in the expression cassettes (Figure 1a), resulting in the exclusion of off‐target integration of the expression cassettes into nuclear or mitochondrial genomes. The homoplasmic lettuce transplastomic lines expressing the native CTB‐FVIII‐C2 sequence (31.0 kDa) were confirmed using specific primer sets including 16sF and 3M. In addition, internal primers annealing to different sequences within the expression cassette (5P, H‐R, L‐F, P‐F, T‐R, Figure 1a) were used to exclude any false‐positive spectinomycin resistant mutants. Therefore, these lines were further evaluated for homoplasmic or heteroplasmic status by Southern blot hybridization.

**Figure 2:**
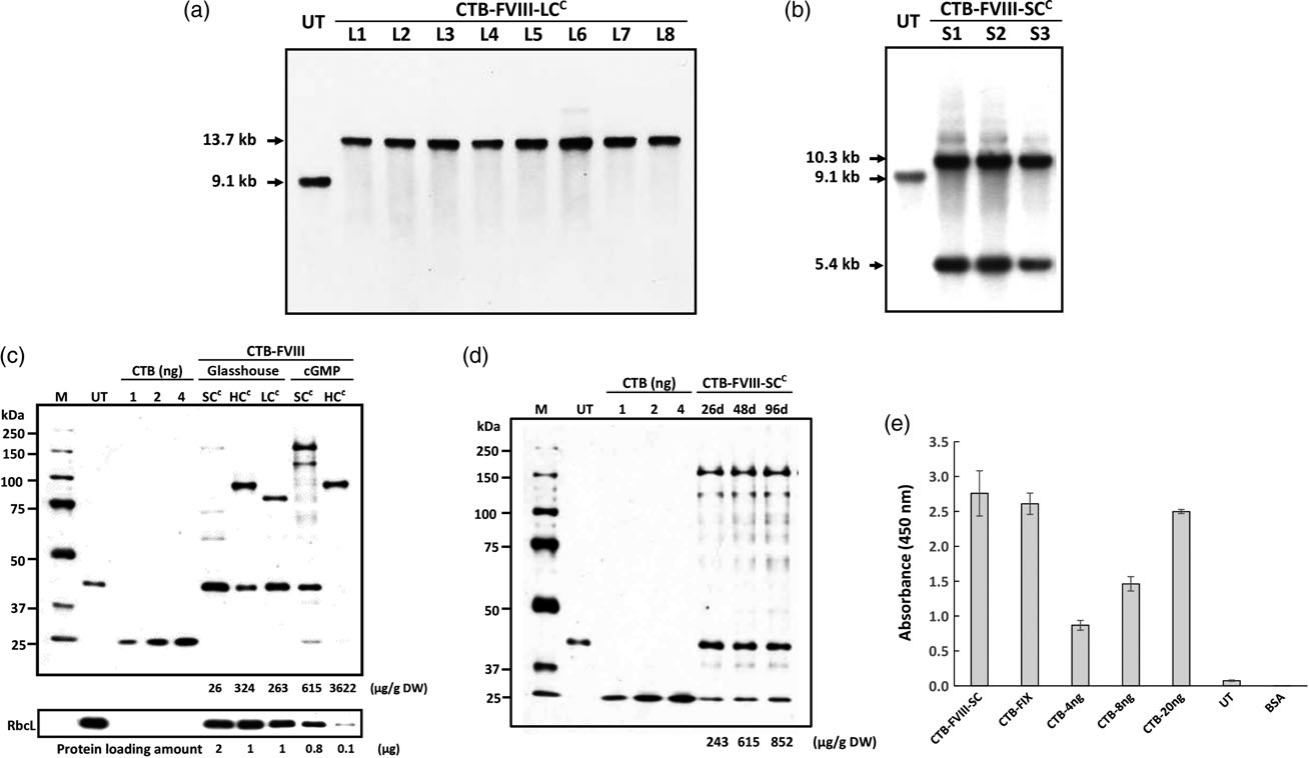
Characterization of CTB‐FVIII transplastomic lettuce lines. (a) and (b) Southern blot analysis: (a) CTB‐FVIII‐LC^C^. UT, untransformed wild‐type DNA; L1–L8, eight independent CTB‐FVIII‐LC^C^ lines. (b) CTB‐FVIII‐SC^C^; S1–S3, three independent CTB‐FVIII‐SC^C^ lines. Total lettuce genomic DNA (3 μg) was digested with *Hind*III. (c) and (d) Western blot analysis of lyophilized lettuce: (c) CTB‐FVIII‐SC^C^ (178.2 kDa), CTB‐FVIII‐HC^C^ (99.3 kDa) and CTB‐FVIII‐LC^C^ (91.8 kDa) expression in soil (glasshouse) or hydroponic (cGMP) systems. (d) Expression level of CTB‐FVIII‐SC^C^ at different stages of growth in the hydroponic system and evaluation of stability of CTB‐FVIII‐SC^C^ protein in lyophilized plant cells after 6‐month storage at ambient temperature. Protein loaded in each lane and expression level based on CTB standards are shown below each lane. UT: untransformed control. (e) GM1 binding assay: CTB‐FVIII‐SC^C^, CTB‐FIX (Herzog *et al*., 2017; Su *et al*., 2015b) and untransformed (200 μg total protein); CTB (4, 8 and 20 ng); 1% BSA. Data shown are means ± SD of triplicates.

For CTB‐FVIII‐LC and CTB‐FVIII‐SC lettuce transplastomic lines, extracted total genomic DNA digested with *Hind*III, and transferred membranes blotted with fragmented genomic DNA were probed with flanking region probe (SB‐P, Figure 1a). In CTB‐FVIII‐LC lettuce lines, the DIG‐labelled probe specifically detected the transformed genomic DNA fragment at 13.7 kb (Figure 2a) with no wild‐type hybridizing fragment at 9.1 kb. In wild‐type lettuce, it only shows the 9.1‐kb DNA fragment. Also, all independent transplastomic CTB‐FVIII‐SC lettuce lines showed two distinct fragments at 10.3 kb and 5.4 kb due to an internal *Hind*III site within the HC coding sequence (Figure 2b), while there was no untransformed wild‐type DNA fragment at 9.1 kb. Absence of 9.1‐kb hybridizing fragment in transplastomic lines confirmed that the homoplasmic lines for CTB‐FVIII‐LC and CTB‐FVIII‐SC were successfully achieved after the 2nd round of antibiotic selection (within limits of this detection system).

### FVIII expression in transplastomic lettuce

For scale‐up of the homoplasmic transplastomic lines expressing CTB‐FVIII‐C2, CTB‐FVIII‐HC, CTB‐FVIII‐LC and CTB‐FVIII‐SC, the confirmed lines were transferred to soil in the glasshouse at the University of Pennsylvania and the cGMP facility in Fraunhofer, USA. The leaves harvested from fully grown lettuce were lyophilized as described in the previous study (Kwon *et al*., 2016). The expressed amounts of CTB‐FVIII‐C2, CTB‐FVIII‐HC, CTB‐FVIII‐LC and CTB‐FVIII‐SC fusion proteins in lyophilized lettuce grown in glasshouse were 367, 324, 263 and 26 μg/g dry weight (DW) of total protein, respectively (Figure 2c). In cGMP hydroponic system, the expressed amounts of CTB‐FVIII‐C2, CTB‐FVIII‐HC and CTB‐FVIII‐SC in the first harvest (day 26) were 3321, 3622 and 243 μg/g DW in lyophilized plant cells. So, there is an increase of 9‐ to 11‐fold higher expression when plants are grown in hydroponic system than on soil in the glasshouse. From Western blot results (Figure 2d), expression level of 26‐day (after seed germination) leaves was 243 μg/g DW; however, the expression level of 48‐day and 96‐day leaves was increased to 615 and 852 μg/g DW, respectively.

For evaluation of stability after long‐term storage, transplastomic lettuce grown in hydroponic system was harvested on days as indicated in Figure 2d and immediately stored in ‐80C deep freezers. Then, different batches were lyophilized on the same day and stored for another 6 months at room temperature. The western blot results give us information on not only the expression levels of each batch but also their stability in lyophilized plant cells when stored at ambient temperature. CTB‐fused FVIII proteins are still intact without any noticeable degradation after 6 months of storage (Figure 2d). Formation of pentameric structures of full‐length CTB‐FVIII was evaluated using GM1‐ganglioside receptor binding ELISA assay. CTB‐FVIII‐SC showed comparable absorbance to CTB and CTB‐FIX (Figure 2e), indicating that it has GM1 binding ability as strong as purified CTB protein and CTB‐FIX clotting factor, despite the large size (891 kDa) of assembled pentamer. This result confirms correct folding, assembly, disulphide bonds and stability of the CTB‐FVIII‐SC pentameric structure in lyophilized leaves.

### Lettuce‐made CTB‐FVIII antigen suppresses inhibitor formation in haemophilia A mice

**Figure 3:**
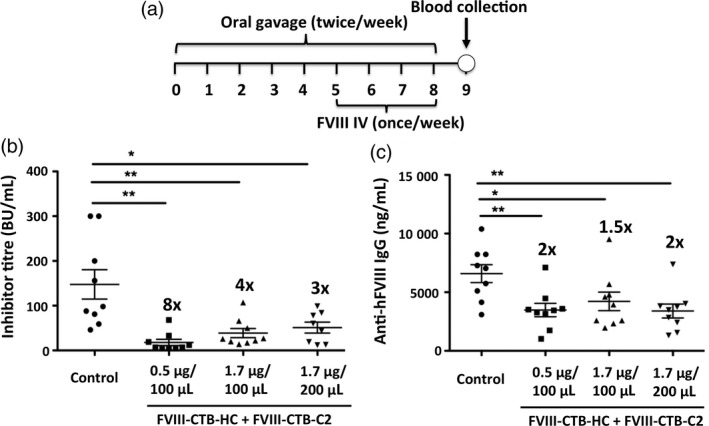
Suppression of inhibitor formation in haemophilia A mice using low doses of FVIII antigens bioencapsulated in lyophilized tobacco. (a) Experimental timeline of oral gavages and intravenous treatment with FVIII. (b) Inhibitor titres in mice that received gavages of 0.5 μg CTB‐HC mixed with 0.5 μg CTB‐C2 in a total volume of 100 μL PBS or 1.7 μg of each antigen in either 100 or 200 μL PBS, compared with control mice (intravenous FVIII challenge only, no oral treatment). (c) FVIII‐specific IgG formation in the same experimental groups. Shown are data point for individual mice, average values and standard deviations. Fold difference and statistical significance are indicated for comparison between each experimental group and the control group (*n* = 8–9/group). Significance is indicated as * for *P*<0.05 and ** for *P*<0.01.

**Figure 4:**
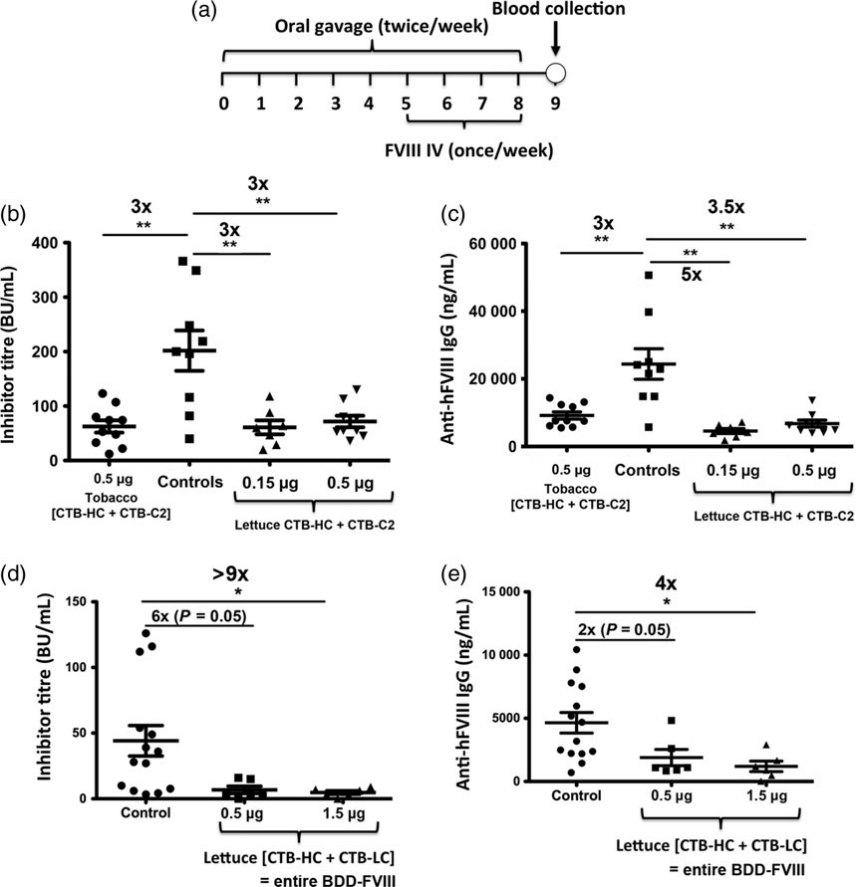
Suppression of inhibitor formation in haemophilia A mice using low doses of CTB‐FVIII‐HC/‐C2 antigens or CTB‐FVIII‐HC/‐LC (containing the entire B domain‐deleted FVIII sequence) bioencapsulated in lyophilized lettuce. (a) Experimental timeline of oral gavages and intravenous treatment with FVIII. (b) Inhibitor titres in mice that received gavages of 0.15 μg CTB‐FVIII‐HC mixed with 0.15 μg CTB‐FVIII‐C2 in a total volume of 100 μL PBS or 0.5 μg of each antigen in 100 μL PBS, compared with control mice (intravenous FVIII challenge only, no oral treatment) and mice that received 0.5 μg of each antigen bioencapsulated in lyophilized tobacco. (c) FVIII‐specific IgG formation in the same HC/C2 experimental groups. Fold difference and statistical significance are indicated for comparison between each experimental group and the control group (*n* = 7–11/group). (d) Inhibitor titres in mice that received gavages of the entire B domain‐deleted FVIII amino acid sequence by mixing 0.5 μg CTB‐FVIII‐HC (heavy chain) with 0.5 μg CTB‐FVIII‐LC (light chain) in a total volume of 100 μL PBS or 1.5 μg of each antigen in 100 μL PBS, compared with control mice (intravenous FVIII challenge only, no oral treatment). (e) FVIII‐specific IgG formation in the same CTB‐FVIII‐HC/‐LC experimental groups. Shown are data point for individual mice, average values and standard deviations. Fold difference and statistical significance are indicated for comparison between each experimental group (*n* = 6/group) and the control group (*n* = 14). Significance is indicated as * for *P*<0.05 and ** for *P*<0.01.

Formation of inhibitory antibodies is a major complication in the clotting factor replacement therapy for haemophilia, as these ‘inhibitors’ prevent the therapeutic coagulation factor from promoting blood clot formation (Batsuli *et al*., 2016). In our previously published study on oral tolerance induction to FVIII (Sherman *et al*., 2014), we demonstrated that codelivery of frozen tobacco cells expressing CTB fusions of HC and C2 domain at a dose of 5 μg per antigen could substantially suppress inhibitor formation. To test for effectiveness of lyophilized cells, which can be stored at ambient temperature for long periods of time, we tested the identical regimen of two oral gavages per week for 2 months in haemophilia A mice, which were challenged with 4 weekly intravenous injections of B domain‐deleted (BDD) human FVIII (1 IU/dose) during the second month of the experiment (Figure 3a). However, substantially lower doses of 0.5–1.7 μg per antigen contained in lyophilized tobacco cells were orally delivered. As shown in Figure 3b, up to eightfold suppression of inhibitor titres (as determined by Bethesda assay; *P *< 0.01, *n* = 9/group) was achieved when compared to control mice that were challenged with intravenous FVIII without having received oral antigen. Suppression of binding antibodies was twofold (as measured by ELISA; *P *<* *0.01, Figure 3c). Next, we directly compared lyophilized tobacco and lettuce for oral tolerance induction using again the combination of CTB‐FVIII‐C2 and CTB‐FVIII‐HC (with lettuce expressing codon‐optimized HC). At doses of 0.5 μg per antigen, both tobacco and lettuce cells were equally effective in suppression of inhibitors and binding antibodies (on average ~3‐fold in this experiment, *P *<* *0.01, *n* = 7–11/group; Figure 4a–c). Interestingly, an even lower dose of 0.15 μg per antigen of lyophilized lettuce was at least as effective, suppressing antibody formation by three‐ to fivefold (Figure 4b,c). Finally, we used the same experimental protocol to test codelivery of lyophilized lettuce expressing CTB fusions of FVIII‐HC and FVIII‐LC (both codon‐optimized), therefore delivering the entire B domain‐deleted FVIII antigen. Here, a dose of 0.5 μg per antigen (*n* = 6) suppressed inhibitor formation on average sixfold and formation of binding antibodies by twofold compared with the control group (*n* = 14), nearly reaching statistical significance (*P *=* *0.05; Figure 4d,e). However, a dose of 1.5 μg per antigen (*n* = 6) significantly and more robustly suppressed inhibitor formation on average 9.3‐fold (with 50% showing 0–3 BU/mL) and formation of binding antibodies by fourfold (*P *<* *0.05; Figure 4d,e). Regardless of FVIII antigen dose, all mice that received oral delivery of CTB‐FVIII‐HC/CTB‐FVIII‐LC had inhibitor titres <25 BU/mL to undetectable (with 50% showing 0–3 BU/mL), while nine of 14 control mice (64%) had titres of >25 BU/mL, and three of 14 (21%) had very high titres of >100 BU/mL, and 100% were >3 BU/mL (Figure 4d).

**Figure 5:**
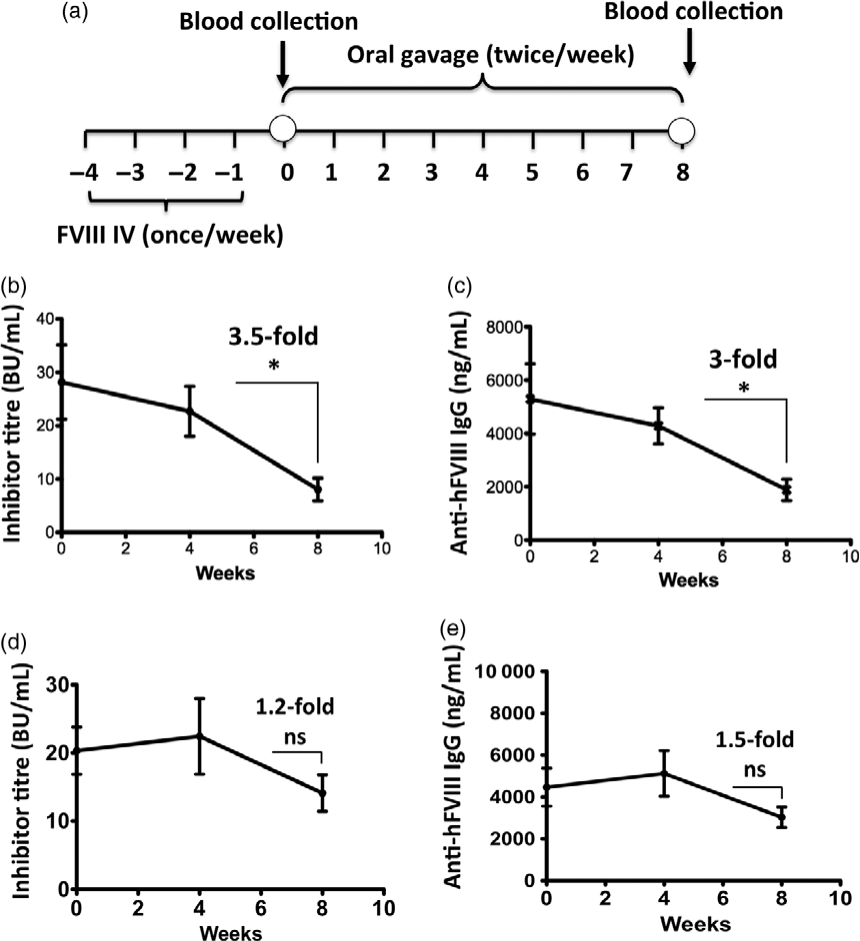
Reversal of inhibitor formation in haemophilia A mice using FVIII antigens bioencapsulated in lyophilized lettuce. (a) Experimental timeline of immunization and oral delivery. (b) Inhibitor titres as a function of time in mice that received gavages of 0.5 μg CTB‐FVIII‐HC mixed with 0.5 μg CTB‐FVIII‐C2. (c) FVIII‐specific IgG formation in the same experimental group (*n* = 13). Fold difference is indicated for comparison between initial and final antibody titres. (d) Inhibitor titres as a function of time in control mice that did not receive oral antigen. (e) FVIII‐specific IgG formation in the same experimental group (*n* = 13). Shown are data point and average values ± standard deviation. Significance is indicated as * for *P*<0.05 and ** for *P*<0.01 (while “ns” is not significant).

While the studies summarized above addressed prevention of inhibitor formation, our previous studies suggested that plant‐based oral tolerance may also be useful in reversal of FVIII inhibitors. To further address this point, we performed the 2‐month oral tolerance regimen, using lyophilized lettuce (CTB‐FVIII‐HC and CTB‐FVIII‐C2 mixture, 0.5 μg/antigen/dose), in haemophilia A mice with pre‐existing inhibitors (average titre of 28 BU, *n* = 13). At the end of the experiment, a 3‐ to 3.5‐fold reduction in anti‐FVIII titres was observed (Figure 5a–c). Inhibitor titres were on average reduced by 71% compared with starting titres (*P *<* *0.05). In contrast, control mice that had an average starting titre of 20 BU (*n* = 9) but did not receive oral treatment showed only a marginal spontaneous decline by <1.5‐fold (31%–32% reduction compared with starting titres, Figure 5d‐e). Therefore, consistent with our previous results using tobacco, oral immune modulatory therapy accelerated the decline in inhibitor titres.

### LAP as a potential biomarker for oral tolerance induction

**Figure 6:**
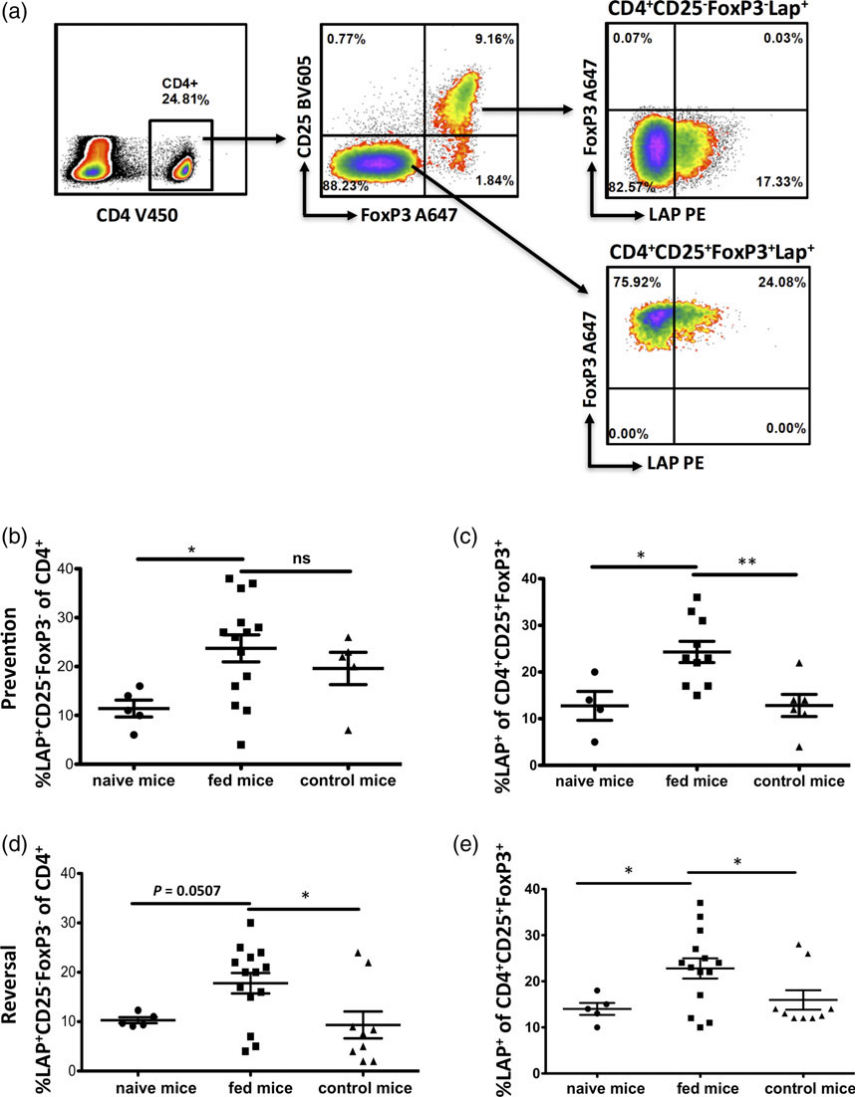
LAP as a potential biomarker expressed by Treg that are induced by oral tolerance. (a) Gating scheme and fluorochrome usage in flow cytometric analysis of peripheral blood cells from haemophilia A mice. (b) Frequencies of LAP^+^CD25^−^FoxP3^−^ Treg among total circulating CD4^+^ T cells after 2 months of oral delivery of CTB‐FVIII‐HC/CTB‐FVIII‐C2 antigen mixture bioencapsulated in lettuce (with weekly intravenous FVIII challenge during the second month, *n* = 14). Comparison between control mice that received intravenous FVIII only (*n* = 5) and naïve mice (*n* = 5). (c) Frequencies of LAP^+^ cells among circulating CD4^+^CD25^+^FoxP3^+^ Treg in the same experimental groups. (d) Frequencies of LAP^+^CD25^−^FoxP3^−^ Treg among total circulating CD4^+^ T cells after 1 month of oral delivery of CTB‐FVIII‐HC/CTB‐FVIII‐C2 antigen mixture bioencapsulated in lettuce in animals with pre‐existing inhibitors (*n* = 14). Comparison between control mice that received intravenous FVIII only (*n* = 9) and naïve mice (*n* = 5). (e) Frequencies of LAP^+^ cells among circulating CD4^+^CD25^+^FoxP3^+^ Treg in the same experimental groups. Shown are data point for individual mice, average values, standard deviations and statistically significant differences.

Previously, we found that plant‐based oral tolerance induces multiple subsets of regulatory T cells (Treg) that suppress antibody formation specifically to the fed antigen. These include CD4^+^CD25^+^FoxP3^+^ Treg, as well as CD4^+^CD25^−^FoxP3^−^ Treg that express latency‐associated peptide (LAP) (Sherman *et al*., 2014; Wang *et al*., 2015). Antigen‐specific CD4^+^CD25^+^FoxP3^+^ Treg typically are present at low frequency, so that it may be difficult to detect their induction by measuring overall frequencies. However, their activation may be detected by up‐regulation of LAP expression. Therefore, we determined the frequency of both LAP^+^CD25^−^FoxP3^−^ cells among circulating CD4^+^ T cells and LAP^+^ cells among circulating CD4^+^CD25^+^FoxP3^+^ cells in haemophilia A mice that received either preventive or interventional oral tolerance (Figure 6a). These were compared to naïve mice or mice that received only intravenous FVIII injections. Depending on the comparison, increase in frequency of LAP^+^CD4^+^CD25^−^FoxP3^−^ Treg did not always reach statistical significance (Figure 6b,d). In contrast, increase in LAP^+^ cells among CD4^+^CD25^+^FoxP3^+^ was consistently statistically significant (Figure 6c,e).

## Discussion

### Codon optimization

To achieve adequate levels of expression of CTB‐FVIII gene suitable for clinical translation, we first codon‐optimized FVIII‐HC and FVIII‐LC separately and verified translation enhancement in *E. coli* because of similarity between *E. coli* and chloroplast transcription/translation systems (Brixey *et al*., 1997). Observed results show that the CTB‐FVIII‐LC expression was increased 15‐ to 42‐fold after codon optimization when compared to the native human gene. In the previous study (Kwon *et al*., 2016), the expression level of CTB‐FVIII‐HC from the codon‐optimized gene was increased eightfold higher than that of lettuce expressing the native CTB‐FVIII‐HC gene. For the carrier protein, the expression level of native CTB fused with proinsulin was 72% of total leaf protein in tobacco chloroplasts (Ruhlman *et al*., 2010) and 53% of total leaf protein in lettuce chloroplasts, which demonstrated that there was no limitation on translational initiation and elongation of N‐terminal CTB sequence in chloroplasts. Hence, the native CTB bacterial sequence was used in this study without codon optimization. Likewise, the native human FVIII‐C2 sequence was used for the expression in lettuce chloroplast because of its high capacity of expression in chloroplast as shown in our previous study (Sherman *et al*., 2014)

### FVIII expression in the homoplasmic lettuce

After confirmation of improved expression of these two separate synthetic genes (HC and LC) in chloroplasts, the two codon‐optimized genes were fused into a single chain (SC) and transplastomic lettuce lines were created. Expression levels of FVIII proteins were not high when grown in the glasshouse in soil because of less‐than‐optimal growth conditions. In addition, clinical translation would require production of biomass in a cGMP facility. Therefore, transplastomic lines were grown in Fraunhofer USA hydroponic facility. The level of CTB‐FVIII‐SC expression of the transplastomic lettuce from hydroponically germinated and grown was ninefold, 24‐fold and 33‐fold higher in the first, second and third harvest than that of soil‐grown lettuce plants. In the first harvest, different FVIII proteins showed nine‐ to 11‐fold higher expression when grown hydroponically than in soil in the glasshouse.

As well documented in the previous studies, increased nitrogen supply enhances plant growth and photosynthetic capacity through the increase of stromal and thylakoid proteins and chloroplast protein synthesis (Evans, 1989; Joel *et al*., 1997; Makino *et al*., 1992). The conversion efficiency of photosynthetically active radiation to biomass was reduced by 30%–35% in nitrogen‐ and water‐limited conditions (Joel *et al*., 1997). Also, as nitrogen supply increases, the ratio of Rubisco to chlorophyll and to electron transport activity was increased (Makino *et al*., 1992). Because Calvin cycle enzymes and thylakoid proteins account for the majority of the leaf nitrogen, the photosynthetic capacity of leaves has a close correlation with the nitrogen content (Evans, 1989). So continuous supply of nitrogen with no water stress in the hydroponic system should have increased the photosynthetic capacity of the lettuce leaves and enhanced chloroplast protein synthesis. Indeed, a previous report has shown that the additional supply of nitrogen is essential to maintain Rubisco levels in chloroplasts when very high levels of foreign proteins are expressed in chloroplasts (Bally *et al*., 2009).

For CTB fusion proteins, the pentameric structure is essential for binding to GM1‐ganglioside receptor on the epithelial cells of intestinal. Even though CTB‐fused FVIII‐SC is the largest protein expressed so far in chloroplasts (pentamer size 891 kDa), the GM1 binding assay confirmed that despite this large size, GM1 binding efficiency is as efficient as CTB or other well‐characterized CTB‐FIX that were successfully tested in haemophilia B dogs (Herzog *et al*., 2017) and mice (Su *et al*., 2015b). Insertion of 10 amino acids in between CTB and FVIII‐SC should help mitigate steric hindrance, otherwise interfering with the proper assembly of pentamer, while forming CTB pentameric structure. Furthermore, this protein is still intact without any detectable cleaved product after 9‐month storage and efficient GM1 binding confirms pentamer assembly with disulphide bonds after 9 months of storage of lyophilized cells at ambient temperature. According to our previous studies (Herzog *et al*., 2017; Su *et al*., 2015b), the CTB‐fused FIX expressed by chloroplast can be stored in lyophilized leaves up to 30 months. Our result confirmed again that the recombinant protein stored in lyophilized plant cell can be preserved for a long time at room temperature without losing their efficacy.

### Suppression of inhibitor formation against FVIII is effective at low antigen doses bioencapsulated in lettuce

In our published studies, we achieved ~7‐fold reduction in average inhibitor titres in haemophilia A mice in response to intravenous FVIII replacement therapy when animals were pretreated orally twice per week with 5 μg CTB‐FVIII‐HC combined with 5 μg CTB‐FVIII‐C2 antigens bioencapsulated in frozen transplastomic tobacco leaf cells. Here, we find up to eightfold suppression using a 10‐fold lower antigen dose in oral delivery of lyophilized cells, which have the advantage of being stable during long‐term storage at ambient temperature (Herzog *et al*., 2017; Su *et al*., 2015b). Therefore, lyophilized cells are equally effective as frozen cells, and consistent with our previous findings with FIX, oral tolerance can be achieved at very low antigen doses. The slightly higher effectiveness of the lowest antigen dose (0.5 μg) over a threefold higher dose in the case of lyophilized tobacco may reflect that in contrast to lettuce, the HC was not codon‐optimized and therefore not as highly expressed, so that a greater amount of plant cells had to be fed, which may not have been entirely digested in the mouse intestine. Previous studies with highly expressed CTB‐FIX in haemophilia B mice showed equal efficacy over a dose range of at least 10‐fold (Su *et al*., 2015b; Verma *et al*., 2010).

Equally important for translational studies in humans, bioencapsulation in the edible crop plant lettuce showed equal efficacy for oral tolerance as tobacco. In fact, an even further reduction in the antigen dose to 0.15 μg, ~30‐fold lower than the original dose in our published tobacco study (Sherman *et al*., 2014), was equally effective. Using LC instead of C2, an adjusted dose of 1.5 μg was superior to a lower dose. The LC is comprised of A3, C1 and C2 domains. The C2 domain is one of the most immunogenic parts of FVIII and contains CD4^+^ T‐cell epitopes in the human population as well as in mice. Furthermore, the C2 domain is considered critical for tolerance induction in haemophilia A mice (Lei and Scott, 2005). As the LC is fourfold larger than the C2 domain, an equal antigen amount represents approximately four times as many CTB‐FVIII‐C2 molecules compared with CTB‐FVIII‐LC. CTB fusions effectively target the GM1 receptor on epithelial cells of the small intestine for effective antigen uptake and transmucosal delivery to the gut immune system. It is possible that the smaller CTB‐C2 antigen may be somewhat more effectively delivered through this mechanism. Nonetheless, we achieved a similar level of suppression of inhibitor formation (eightfold) using a ~3‐fold lower antigen dose as previously published (Sherman *et al*., 2014) with the HC/LC antigen combination. While it is likely not necessary to cover all CD4^+^ T‐cell epitopes with the orally delivered antigen in an individual person (because induced Treg should suppress responses to other parts of the molecule, Kim *et al*., 2015), the human population is diverse with regard to MHC II genes and T‐cell epitope usage. Therefore, in some patients, inclusion of the C1 domain may be more critical than the C2 domain. Hence, coverage of the entire molecule for oral antigen delivery should be most beneficial for treatment of the human patient population. In summary, we accomplished multiple critical steps towards oral tolerance induction to FVIII in haemophilia A patients. Specifically, we showed effectiveness of low antigen doses in leaf cells (prepared in a form that is ideal for stable long‐term storage) of a crop plant that is suitable for human application, and we covered the entire antigen. Low antigen doses both reduce manufacturing costs and facilitate administration and dose‐finding studies in humans.

Reversal of an established immune response is typically more difficult than prevention. However, our results support a utility in oral tolerance in inhibitor reversal as well. Currently, inhibitor patients are subjected to ITI (immune tolerance induction) protocols, which typically involve high‐dose daily intravenous infusion of FVIII, which can take months and is very expensive. In addition, these protocols are more effective when initiated after inhibitor titres spontaneously declined to lower levels. This period of time adds to the duration of increased risks of morbidity and mortality. Certain types of immune suppression may be an alternative (Biswas *et al*., 2017). However, our new data with lettuce and published results with tobacco reproducibly show that plant‐based oral tolerance can accelerate the decline of pre‐existing inhibitors and could therefore be incorporated into future ITI protocols (Sherman *et al*., 2014). Reversal of FIX inhibitors and of anaphylaxis in haemophilia B mice further support a role for oral tolerance in reversal of immune responses in the treatment of haemophilia (Wang *et al*., 2015).

### Towards cellular biomarkers of oral tolerance induction

Translational oral tolerance studies in humans are complicated by the variability of the response to FVIII. It would therefore be helpful to include a panel of biomarkers to clinical trial design. In previous studies, we found that oral antigen delivery followed by challenge with intravenous antigen induced two subsets of Treg that suppressed antibody formation against FVIII or FIX systemically. These were CD4^+^CD25^+^FoxP3^+^ Treg and LAP^+^CD4^+^CD25^−^FoxP3^−^ Treg. The latter were most robustly induced in the gut immune system (e.g. in Peyer's patches and mesenteric lymph nodes), but were also detectable in the spleen (Sherman *et al*., 2014; Wang *et al*., 2015). LAP^+^ Treg overexpress the immune‐suppressive cytokine TGF‐β, and their ability to suppress immune responses is also TGF‐β‐dependent (Ochi *et al*., 2006). Here, we also find increases in the frequencies of these cells in peripheral blood, albeit a significant increase was not always observed when compared to the blood of control mice that received intravenous FVIII but not oral tolerance. Antigen‐specific CD4^+^CD25^+^FoxP3^+^ Treg tend to suppress at low frequencies, so that we did not observe significant increases in their overall frequency, but were able to demonstrate their induction by adoptive transfer (Sherman *et al*., 2014; Wang *et al*., 2015). However, their activation may result in induction of LAP expression on the cell surface (Tran *et al*., 2009a,b). Our new data show significant increases in the frequencies of LAP^+^CD4^+^CD25^+^FoxP3^+^ Treg in peripheral blood of tolerized mice, suggesting that LAP, and in particular this subset of Treg, may be a suitable cellular biomarker for plant‐based oral tolerance induction. Importantly, the human immune system also contains LAP^+^ Treg (Gandhi *et al*., 2010).

### Chloroplast expression system

Our study achieved expression of the highly challenging large human FVIII single‐chain protein (pentamer size 891 kDa) – the largest foreign protein ever expressed in chloroplasts. The amino acid sequence of FVIII‐SC studied here is the same as that of ReFacto^®^ or ReFacto AF^®^, which are currently used in clinics as replacement therapeutic FVIII products (Lind *et al*., 1995; Orlova *et al*., 2013). Although efficacy of CTB‐FVIII single chain has not yet been fully investigated in animal studies, a single candidate protein target should offer several advantages. First, it meets the clinical need because it covers the entire patient population, regardless of individual CD4^+^ T‐cell epitope responses. Secondly, regulatory approval cost is significantly reduced for a single protein versus two different protein targets. Because the light chain and heavy chain are equal in dosage and efficacy, there is no need to change their ratios while administering this drug. Most importantly, the negative impact of the light chain (slow growth, sterility) is mitigated by fusion with the heavy chain. As seen in our previous report (Su *et al*., 2015a), the phenotypes observed from LC lettuce could be a result of a deleterious effect from the binding of the LC with an essential chloroplast protein. Transplastomic plants expressing CTB‐fused truncated form of acid alpha glucosidase (410 aa of N‐terminal GAA) showed yellow or albino phenotype and full‐length GAA could never be expressed in chloroplasts (Su *et al*., 2015a). This was due to a possible disruption of normal metabolism in chloroplast as a consequence of the binding of the truncated GAA to mannose 6‐phosphate receptor or the homologue in the chloroplast (Su *et al*., 2015a). Another possible explanation could be inferred from a recent study, which showed that the LC itself is sufficient for accelerating cleavage of von Willebrand factor (VWF) by metalloprotease (A disintegrin and metalloprotease with thrombospondin type 1 repeats, ADAMTS13) (Cao *et al*., 2012). So, the LC could probably impact chloroplast proteases, growth of transplastomic lines and fertility. However, it appears that the fusion of HC with LC minimized detrimental impact of the LC.

This is the first report of animal studies expressing FVIII‐HC, FVIII‐C2 and FVIII‐LC made in lettuce chloroplasts. Combination of FVIII‐HC/FVIII‐LC, spanning the entire FVIII antigen, has never before been evaluated in tobacco or lettuce. Furthermore, we compared efficacy of transplastomic tobacco and lettuce because there is still hesitation among investigators in this field regarding reproducibility of an edible system when compared to a model system on critical requirements including level of transgene expression, differences in bioencapsulation to protect protein drugs in the stomach, etc. Likewise, previous studies used frozen tobacco cells for oral gavage, whereas this study used lyophilized lettuce cells, which facilitates long‐term storage at ambient temperature and is critical for clinical feasibility. We are delighted to report here our observation that the lyophilized lettuce system is as efficient as the frozen tobacco system at very low dose of plant material (≤2.25 mg versus 125 mg per gavage). Therefore, lyophilized lettuce FVIII can be advanced to the clinic.

This study achieved a high level of expression of a large human FVIII single‐chain protein, the largest foreign protein ever expressed in chloroplasts. This study demonstrates that chloroplast expression system can indeed be further optimized to meet clinical needs by enhancing translation through codon optimization, utilizing recent advances in genomics and genetic engineering. Although biomass is produced in controlled facilities, unintentional dispersal of human genes is always a critical concern of genetically modified plants. Therefore, maternal inheritance of chloroplast genome offers transgene containment via pollen or seeds because biomass is harvested before appearance of reproductive structures (Daniell, 2007; Daniell *et al*., 2016a; Jin and Daniell, 2015). Most importantly, therapeutic proteins expressed in chloroplasts can be stably stored at ambient temperature for several years without decrease in their functionality (Herzog *et al*., 2017; Su *et al*., 2015b). In this study, we showed that an antigen combination that covers the entire amino acid sequence of BDD‐FVIII (HC and LC), one of the commonly used therapeutic versions of FVIII, is effective. Oral delivery of therapeutic proteins bioencapsulated in plant cells significantly reduces cost of biopharmaceuticals by elimination of expensive fermentation, purification, cold storage and sterile injections. Therefore, CTB‐FVIII expressed in lettuce chloroplasts will benefit patients with increased compliance, lower cost and induction of immune tolerance after receiving FVIII injections.

## Materials and methods

### Codon optimization of human FVIII genes and construction of chloroplast vectors

The native sequences of FVIII genes were codon‐optimized, except for C2, based on the codon table which was developed by analysis of *psbA* genes from 133 plant species (Kwon *et al*., 2016). The eukaryotic genes were optimized by adapting the codon usage hierarchy of the most highly expressed chloroplast *psbA* gene. The codon‐optimized FVIII‐HC and FVIII‐LC sequences were synthesized by GenScript, and the codon‐optimized FVIII‐SC was generated by overlapping PCR using the codon‐optimized FVIII‐HC and FVIII‐LC as templates. For FVIII‐C2, the native C2 gene fragment was obtained from the previous construct, CTB‐FVIII‐C2:pLD (Sherman *et al*., 2014), by digestion with restriction enzymes. The synthetic and the native C2 gene sequences were cloned into lettuce chloroplast transformation vectors pLsLF using *Nde*I and *Xba*I restriction enzyme sites. Sequence‐confirmed plasmids were transformed into TOP10 *E. coli* cell and extracted using PureYield™ Plasmid Midiprep System (Promega, Madison, WI).

For evaluation of the protein expression level in *E. coli* system, overnight cultured *E. coli* cells in ampicillin‐containing LB medium were pelleted, resuspended in extraction buffer (1 × PBS and 5 mm EDTA) and then sonicated to break down cells. The homogenate of *E. coli* total protein was quantified using Bradford assay, and equal amount of proteins was loaded after heating samples at 70 °C for 15 min in 1 × Laemmli buffer.

### Generation of transplastomic lettuce

Three lettuce expression vectors pLsLF‐CTB‐FVIII‐SC^C^, pLsLF‐CTB‐FVIII‐HC^C^ and pLsLF‐CTB‐FVIII‐LC^C^ (Figure 1a) were transformed into 4‐week‐old lettuce (*Lactuca sativa*) leaves by particle bombardment using 0.6‐μm gold particle (Bio‐Rad, Hercules, CA) in 900 psi (Verma *et al*., 2008). The bombarded leaves were subject to multiple selection rounds in selection/regeneration medium with spectinomycin (50 μg/mL). The regenerated shoots were examined by PCR amplification and Southern blot assay using specific primer sets and probes (Figure 1a). The sequences of primers used for the PCR analysis are as below: 16sF, 5΄‐ CAGCAGCCGCGGTAATACAGAGGATGCAAGC‐3΄; 5P, 5΄‐CTGTAGAAGTCACCATTGT TGTGCACGACGAC‐3΄; 3M, 5΄‐ CCGCGTTGTTTCATCAAGCCTTACGGTCACC‐3΄; P‐F, 5΄‐ ACAGCTGTCGACTAGTGTATAGAAA TCCTT‐3΄; H‐R, 5΄‐AGACATAATGGATCGGA GGCCATAGGTCC G‐3΄; L‐F, 5΄‐ACTACATTGCTGCAGAAGAAGAGGATTGGGATTATG C‐3΄; T‐R, 5΄‐GCAAGAGCGGA GCTCTACCAACTGAGCT A‐3΄. Southern blot analysis for checking homoplasmic status of transplastomic lines was examined by following the manufacturer's instruction of DIG high prime DNA labelling and detection starter kit II (Roche, Penzberg, Germany).

To generate a large scale of lettuce biomass, the transplastomic lettuces were grown in two different cultivation systems, the glasshouse (soil system) and cGMP facility (hydroponic system). Seedling stage plants grown in in vitro culture system were transferred onto soil in the glasshouse. The soil was mixed with a 1 : 1 ratio of garden soil (Miracle‐Gro) and potting soil (Erthgro), and the plants were fertilized with Miracle‐Gro Water Soluble All Purpose Plant Food once or twice per week under the conditions of 16‐h : 8‐h light cycle at 22 °C. Cultivation of the lettuce in hydroponic system was described in our previous study (Su *et al*., 2015b). The hydroponic system included multilevel growth racks which were illuminated by three sealed lighting fixtures containing 2‐T8 fluorescent bulbs. Each rack accommodated two growth trays containing rockwool fibre as supporting substrates (Grodan, the Netherlands) which are designed to hold water longer and encourage faster initial rooting by providing 20% airspace while absorbing nutrients when saturated. The nutrient with a 20‐10‐20 ratio of nitrogen, phosphorus and potassium was supplied when irrigated. Seedlings were spaced at 2 inch × 2 inch on the rockwool surface. Plants were exposed to light of an average of 70–90 μmol/m^2^/s on an 18‐h photoperiod under the conditions of temperature (23–26 °C) and humidity (20%–60%) in the growth room. In cGMP hydroponic system, the transplastomic lettuces were harvested in day 26, day 48 and day 96. After sample harvested, lyophilization and preparation of powder of lettuce leaves were carried out as described previously (Kwon *et al*., 2016).

### Total protein extraction and quantification

To evaluate the level of expression of the synthetic FVIII genes in transplastomic lettuce, the harvested fresh lettuce leaves from glasshouse or hydroponic systems were lyophilized and powdered and then suspended in plant extraction buffer (100 mm NaCl; 10 mm EDTA; 200 mm Tris‐Cl, pH 8.0; 0.05% (v/v) Tween‐20; 0.1% SDS; 14 mm β‐ME; 400 mm sucrose; 2 mm PMSF; 1 × proteinase inhibitor cocktail) in a ratio of 10 mg per 500 μL and rehydrated with intermittent vortex in 4 °C. The rehydrated plant cells were sonicated in pulse on for 5 s and pulse off for 10 s by sonicator 3000 (Misonix, Farmingdale, NY). After protein quantification by Bradford assay, equal amounts of homogenized proteins were heated at 70 °C in 1 × Laemmli buffer for 15 min.

SDS‐polyacrylamide page (8%, fixed) was used for Western blots. To probe CTB fusion proteins, anti‐CTB antibody (1 : 10000) (GenWay Biotech, San Diego, CA) and goat anti‐rabbit IgG‐HRP secondary antibody (1 : 4000) (Southern Biotechnology, Birmingham, AL) were used. Chemiluminescent substrate (Thermo Fisher, Waltham, MA) was added for detecting HRP on the immunoblots, and the signals were exposed onto X‐ray films, and then, developed bands were used for quantitative analysis with ImageJ software (IJ 1.46r; NIH).

### GM1‐ganglioside receptor binding assay

To evaluate the ability of the lettuce chloroplast‐derived CTB‐FVIII to form pentamers and bind to the GM1‐ganglioside receptor, a CTB‐GM1 binding assay was performed. For CTB‐FVIII protein extraction, lyophilized plant powder was suspended in extraction buffer in a ratio of 10 mg per 500 μL and mixed by vortexing for 1 h at 4 °C for rehydration. Suspended powder was sonicated (pulse on for 5 s and pulse off for 10 s; sonicator 3000; Misonix) after vortexing. CTB‐GM1 binding assay was carried out by following the protocol described previously (Ruhlman *et al*., 2007).

### Haemophilia A mouse experiments

Male haemophilia A mice (F8e16^−/−^) on BALB/c background, 2 months of age at the onset of experiments and housed under SPF conditions at University of Florida CGRC facility, received oral gavage of mixture CTB‐FVIII‐C2 and CTB‐FVIII‐HC lyophilized tobacco or lettuce leaf cells (suspended in 100–200 μL of sterile phosphate‐buffered solution) twice per week for 2 months. At the beginning of the second month, mice received weekly intravenous injection of 1 IU/mouse of recombinant BDD‐FVIII concentrate (Xyntha, Prizer, New York, NY), for a total of four injections. One week after last IV injection, blood was collected via retro‐orbital route into citrated buffer, and blood cells were removed by centrifugation. Plasma was and assessed for inhibitors (by Bethesda assay) and for FVIII‐specific IgG (by ELISA) as published (Sherman *et al*., 2014).

### Flow cytometry

Murine blood samples were pre‐incubated with rat anti‐mouse CD16/32 (Pharmingen, Franklin Lakes, NJ) and then stained with a mixture of eFluor 450 CD4 (eBioscience, Waltham, MA), BV605, anti‐mouse CD25 (Biolegend, San Diego, CA) and PE anti‐mouse LAP (Biolegend). Intracellular staining for forkhead box P3 (FoxP3) was performed with FoxP3 staining kit (eBioscience). Data were collected on LSR II flow cytometer (BD Bioscience, San Jose, CA) and analysed with FCS express software (De Novo Software, Glendale, CA).

### Statistical analysis

Differences between two experimental groups were compared by unpaired two‐tailed Student's *t*‐test. Results are reported as means ± standard error of mean (SEM). Differences are indicated as *P <* *0.05, **P <* *0.01 and ns = not significant. GraphPad Prism software (San Diego, CA) was used to perform analysis.

## Conflict of interest

Although there is no financial conflict of interest to report, the corresponding author is an inventor on numerous patents reporting expression of human therapeutic proteins in chloroplasts. For a complete list of patents, please see Google Scholar link provided below. Partial funding for this research and travel support was provided by Novo Nordisk. http://scholar.google.com/citations?user=7sow4jwAAAAJ&hl=en.

## Supporting information


**Figure S1** Sequences of recombinant human FVIII single chain.
