# Supplementary figures and images for: Expression and assembly of largest foreign protein in chloroplasts: oral delivery of human FVIII made in lettuce chloroplasts robustly suppresses inhibitor formation in haemophilia A mice
Source: Plant Biotechnol J. 2017 Dec 7;16(6):1148–60. doi: 10.1111/pbi.12859 (PMC5936678; doi:10.1111/pbi.12859)

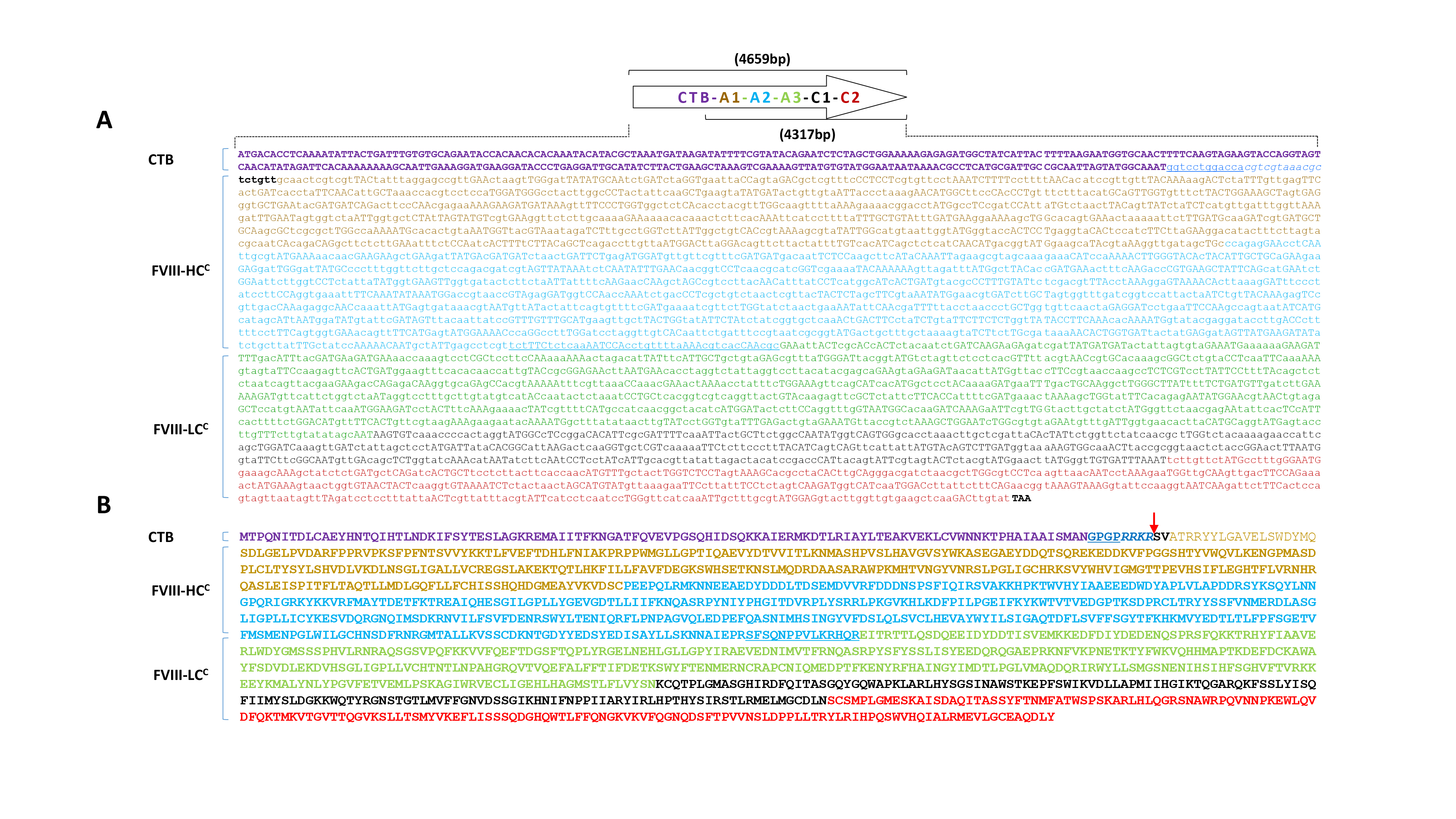

Supplement: Supplementary file 1 — Figure S1 Sequences of recombinant human FVIII single chain. [file PBI-16-1148-s001.jpg]
